# The impact of chronic obstructive pulmonary disease and obesity on length of stay and cost of spine surgery

**DOI:** 10.4103/0019-5413.67120

**Published:** 2010

**Authors:** M Sami Walid, Nadezhda V Zaytseva

**Affiliations:** Medical Center of Central Georgia, Macon, GA, USA; 1 Kuban State Medical University, Krasnodar, Russia

**Keywords:** COPD, obesity, spine surgery, length of stay, hospital cost

## Abstract

**Background:**

Chronic obstructive pulmonary disease (COPD) and obesity may be more common among spine surgery patients than in the general population and may affect hospital cost.

**Materials and Methods:**

We retrospectively studied the prevalence of COPD and obesity among 605 randomly selected spine surgery inpatients operated between 2005 and 2008, including lumbar microdiskectomy, anterior cervical decompression and fusion and lumbar decompression and fusion patients. The length of hospital stay and hospital charges for patients with and without COPD and obesity (body mass index [BMI]≥30 kg/m^2^) were compared.

**Results:**

Among 605 spine surgery patients, 9.6% had a history of COPD. There were no statistical difference in the prevalence of COPD between the three spine surgery groups. Obesity was common, with 47.4% of the patients having a BMI≥30 kg/m^2^. There were no significant differences in obesity rates or BMI values between the three types of spine surgery patients. Obesity rates between patients with and without COPD were 62.1% vs. 45.9%, and were statistically different (*P*<0.05). Similarly, significant difference (*P*<0.01) in BMI values between COPD and non-COPD groups, 32.66±7.19 vs. 29.57±6.048 (mean ± std. deviation), was noted. There was significant difference (*P*<0.01) in cost between nonobese female patients without COPD and those with obesity and COPD in the anterior cervical decompression and fusion (ACDF) group. No association with increased hospital length of stay or cost was found in the other two types of spine surgery or in male ACDF patients.

**Conclusion:**

COPD and obesity seem to additively increase the length of hospital stay and hospital charges in ACDF female patients, an important finding that requires further investigation.

## INTRODUCTION

Chronic obstructive pulmonary disease (COPD) refers to a group of three lung diseases – chronic bronchitis, asthma and emphysema – which frequently blend together in a chronic lung disease that worsens over time with intermittent exacerbations. COPD is the fourth leading cause of death in the USA, behind heart disease, cancer and stroke, killing more than 120,000 Americans each year.^1^ More than 12 million people are currently diagnosed with COPD and an additional 12 million are estimated to have a mild form of the disease.^1^,^2^ It is also a major cause of serious long-term disability.^1^ Excess healthcare expenditures are estimated at nearly $6,000 annually for every COPD patient.^3^ The economic burden of COPD in 2007 was estimated at $42.6 billion in treatment cost and lost productivity.^4^

In addition to COPD, 32.2% of adult American men and 35.5% of adult American women are obese.^5^ In the literature, a relationship between COPD and obesity is increasingly recognized but still “undervalued.”^6^,^7^ Pursuant to this, we thought that spine surgery patients with COPD and obesity may be more common than in the general population and would consume higher healthcare resources. Given the current economic crisis and the financial difficulties the US healthcare system is facing, it is important to scrutinize any factors that may influence healthcare cost. Spine surgery is a good example of a common and expensive surgery.^14^ More back surgeries are performed in the US than in other countries, and the number is expected to increase with the quick advancements in the surgical field.^15^ In this paper, we studied the prevalence of COPD and obesity among spine surgery patients and their impact on length of stay and hospital cost.

## MATERIALS AND METHODS

We reviewed 605 randomly selected spine surgery inpatients who were operated between 2005 and 2008 to study the prevalence and economic impact of COPD and obesity in spine surgery. The type of surgery, history of COPD (asthma, emphysema and/or chronic bronchitis), body mass index (BMI), length of stay and hospital cost (charges) were entered in a data excel file. Three types of spine surgery were included: lumbar microdiskectomy (*n*=199), anterior cervical decompression and fusion (*n*=244) and lumbar decompression and fusion (*n*=162). The median age of patients were 54 years (range 14-89 years). Patients were 45% males and 55% females, and 73.2% Caucasians vs. 25.5% African Americans. We calculated the percentages of patients with already known COPD and obesity (BMI≥30 kg/m^2^). Using SPSS v.16, we applied chi-square test to compare percentages and analysis of variance (ANOVA) to check for significant differences in length of stay and hospital cost.

## RESULTS

The 605 spine surgery patients had a median BMI of 29 kg/m^2^ (range 17–58 kg/m^2^). Among the 605 spine surgery patients, 9.6% had a history of COPD [Table 1]. Using chi-square analysis, no statistical differences between the three types of surgery regarding the prevalence of COPD were noted.

**Table 1:** Distribution of spine surgery patients per COPD status

	Type of spine surgery					
	LMD		ACDF		LDF	
	No.	Percentage	No.	Percentage	No.	Percentage
No COPD	181	91.0	224	91.8	142	87.7
COPD	18	9.0	20	8.2	20	12.3
Chi-square	2.034
df	2
Sig.	0.362

LMD = Lumbar microdiskectomy, ACDR = Anteriorcervical decompression and fusion, LDF = Lumbar decompression and fusion, df = difference, Sig = Significance

**Figure 1:**
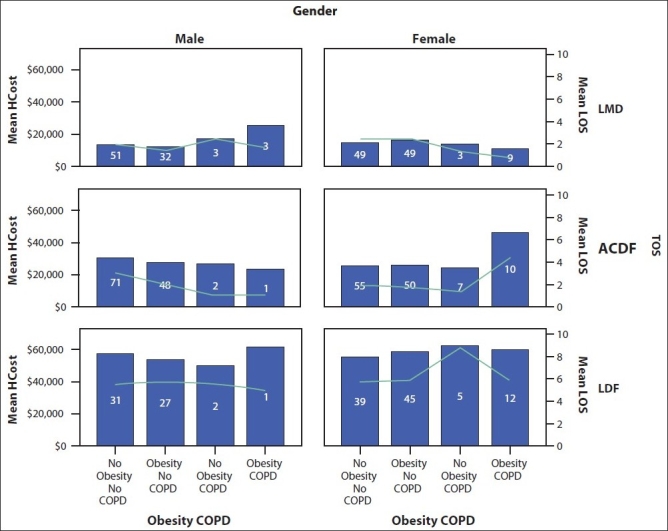
Bar diagram showing length of stay and hospital cost per gender, type of spine surgery, obesity and COPD status with the number of cases in each category (inside the bars)

Obesity was common, with 47.4% of patients having a BMI≥30 kg/m^2^. There were no significant differences in obesity rates (nominal variable) or BMI values (scale variable) among the three types of spine surgery. Obesity rates among patients with and without COPD were 62.1% vs. 45.9%, and were statistically different (*P*<0.05) in addition to a significant difference (*P*<0.01) in BMI values among the COPD and non-COPD groups, 32.66±7.19 vs. 29.57±6.048 (mean ± std. deviation). COPD and obesity correlated significantly (r=0.09, *P*<0.05). We studied the impact of COPD and obesity coexistence on length of stay and hospital cost and found that they have an additive (and probably synergistic) effect in the female ACDF group only, nearly doubling both length of stay and cost [Table 2]. There was a significant difference in hospital cost (*P*<0.01) between patients with no obesity or COPD and those with obesity and COPD in the ACDF female group [Figure 1].

**Table 2:** Length of stay and hospital cost per gender, type of spine surgery, COPD and obesity

	Male			Female		
	LMD Mean	ACDF Mean	LDF Mean	LMD Mean	ACDF Mean	LDF Mean
No obesity, no COPD						
LOS	2	3	6	2	2	6
Hcost ($)	14,322	30,876	58,085	15,309	25,517	56,132
Obesity, no COPD						
LOS	1	2	6	2	2	6
Hcost ($)	13,056	27,676	54,346	16,866	26,290	59,580
No obesity, COPD						
LOS	2	1	6	1	1	9
Hcost ($)	17,759	27,238	50,436	14,291	24,581	63,068
Obesity, COPD						
LOS	2	1	5	1	4	6
Hcost ($)	25,896	23,407	62,457	11,322	46,478	60,658

LOS = Length of stay in days, Hcost = Hospital cost, LMD = Lumbar microdiskectomy, ACDR = Anteriorcervical decompression and fusion, LDF = Lumbar decompression and fusion

## DISCUSSION

Higher complication rate among COPD patients undergoing different types of surgery eg. pulmonary, abdominal, cardiovascular, orthopedic, etc. have been reported.^8^–^16^ Our paper is the first study that addresses COPD as a medical comorbidity in spine surgery. In chronic back pain patients, COPD and obesity act as an annoying vicious cycle where COPD can lead to obesity via sedentary life or as a side-effect of treatment with corticosteroids then obesity itself worsens COPD tolerance and back pain problems. We found that COPD in combination with obesity significantly increases hospital length of stay and cost for cervical spine surgery is female patients. This is an interesting finding that can be related to the proximity of the operative site to the upper airways. Traditionally, the diagnosis of COPD has not been considered an absolute contraindication to surgery. However, patients with COPD are well known to be at higher risk for perioperative complications. First, problems with ventilation can be life-threatening. The COPD patient may be more sensitive to the ventilatory depressant effects of analgesic and anesthetic agents. Therefore, the immediate postoperative recovery period for COPD patients requires close monitoring for respiratory muscle dysfunction, acidemia, hypoxaemia and hypoventilation, especially if associated with obesity.^10^,^15^,^17^ One of the possible explanations for the above findings in the ACDF group could be postoperative edema around the upper airways, with an aggravating effect from comorbid COPD and obesity. This effect was not seen in the lumbar surgery groups, suggesting that pulmonary complications after spine surgery depend on the distance of the procedure site from the upper airways. COPD patients also have an increased risk of postoperative infection, including pneumonia and wound infection.^8^,^9^,^11^,^15^–^16^ This is why postoperative antibiotics are unhesitantly indicated for these patients.^18^ In addition, patients with COPD have lower tolerability of heart arrhythmias and pulmonary embolism and, if they happen, are more likely to be fatal.^14^,^19^ COPD has as well been associated with osteoporosis, which necessitates more caution during early postoperative ambulation and rehabilitation.^20^,^21^

The dissimilarity in the impact of COPD and obesity on length of hospital stay and cost in cervical spine surgery in males and females can be referred to gender differences in COPD prognosis and BMI connotation as a measure of obesity. Females have been reported to have a worse prognosis of COPD and higher measures of adiposity.^22^,^23^ BMI, used in our study, does not differentiate between obese men and muscular men, which may skew the results in the male cohort.^24^ Moreover, the discovered association of COPD and obesity with increased length of stay and hospital cost in the cervical variant of spine surgery may not be true for hospital systems that have different structure and payment arrangements from the American healthcare system.

We conclude that the identification of COPD as comorbidity should be an essential part of the preoperative visit of ACDF candidates, especially obese females. Scrupulous case finding among patients with clinical signs or symptoms suggestive of COPD can exclude risky subjects and increase cost-effectiveness of the neurosurgical practice. Further research is needed into the possible beneficial role of pulmonary consultation and pulmonary function testing in diagnosing and optimizing breathing parameters before ACDF surgery.
